# Clinical experience of repair of pectus excavatum and carinatum deformities

**DOI:** 10.5830/CVJA-2013-065

**Published:** 2013-10

**Authors:** Murat Oncel, Güven Sadi Sunam, Bekir Tezcan, Kazim Gurol Akyol, Yüksel Dereli

**Affiliations:** Department of Thoracic Surgery, Faculty of Medicine, Selcuk University, Konya, Turkey; Department of Thoracic Surgery, Faculty of Medicine, Selcuk University, Konya, Turkey; Department of Thoracic Surgery, Konya State Hospital, Konya, Turkey; Department of Thoracic Surgery, Konya State Hospital, Konya, Turkey; Department of Cardiovascular Surgery, Faculty of Medicine, Necmettin Erbakan University, Konya, Turkey

**Keywords:** pectus excavatum, pectus carinatum, modified Ravitch procedure, internal bar

## Abstract

**Background:**

We present the results of surgical correction of pectus excavatum (PE) and pectus carinatum (PC) deformities in adults, and also report a new method of sternal support used in surgery for PE deformities.

**Methods:**

We present the results of 77 patients between the ages of 10 and 29 years (mean 17) with PE (*n* = 46) or PC (*n* = 31) deformities undergoing corrective surgery from 2004 to 2011, using the Ravitch repair method. Symptoms of the patients included chest pain (15%) and tachycardia (8%). Three patients underwent repair of recurrent surgical conditions.

**Results:**

All of the patients with dyspnoea with exercise experienced marked improvement at five months post operation. Complications included pneumothorax in 5.1% (*n* = 4), haemothorax in 2.6% (*n* = 2), chest discomfort in 57% (*n* = 44), pleural effusion in 2.6% (*n* = 2), and sternal hypertrophic scar in 27% (*n* = 21) of patients. Mean hospitalisation was eight days. Pain was mild and intravenous analgesics were used for a mean of four days. There were no deaths. Results after surgical correction were very good or excellent in 62 patients (80%) at a mean follow up of three years. Three patients had recurrent PE and were repaired with the Nuss procedure. In three patients who underwent the Ravitch procedure, a stainless steel bar was used for sternal support instead of Kirschner wire.

**Conclusions:**

Pectus deformities may be repaired with no mortality, low morbidity, very good cosmetic results and improvement in cardiological and respiratory symptoms.

## Abstract

Pectus excavatum (PE) may be as common as one in 300 live births.^1^ It is usually noticed within the first year of life. Sternal depression becomes much more pronounced in early adolescence during rapid skeletal growth. PE is well tolerated in infancy but adult patients may suffer from poor chest movement in areas of the deformed cartilage, often resulting in serious exercise intolerance. The heart is often displaced to the left chest by the depressed sternum. Posterior displacement of the sternum can produce a deformity of the heart, specifically the anterior of the right ventricle.^2^ Exercise tolerance is usually improved after operation for PE.

Pectus carinatum (PC) is the best recognised and most frequent protrusion deformity of the chest wall.^1^ PC is usually identified up to the 11th birthday. Mixed deformities have a carinate deformity on one side and a depression or excavatum deformity on the contralateral side.

This study aimed to determine the results of surgical correction of PE and PC deformities in adults. We also report a new method of sternal support used in surgery for PE deformities.

## Methods

**Fig. 1.**
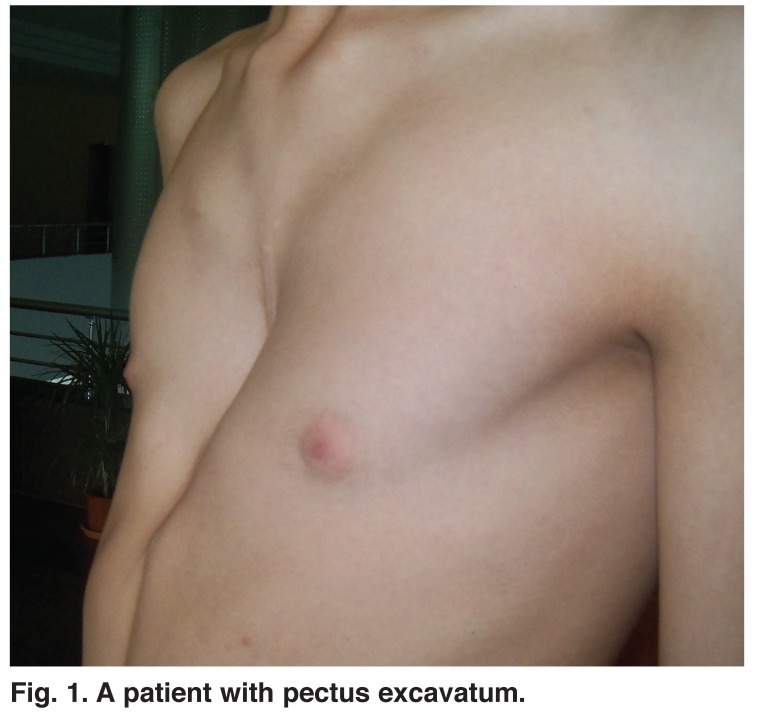
A patient with pectus excavatum.

**Fig. 2.**
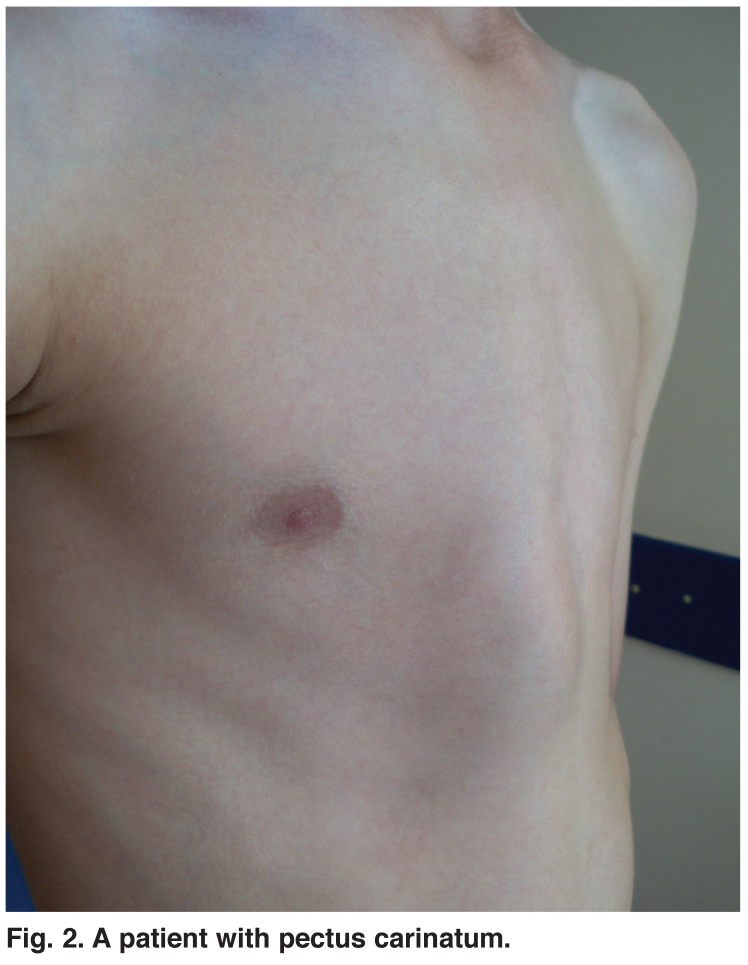
A patient with pectus carinatum.

Patients between the ages of 10 and 22 years who underwent correction of PE and PC deformities at the Konya State Hospital and Faculty of Medicine, Selcuk University, between January 2004 and December 2010, were reviewed (Figs 1, 2). Indications for the operation included Haller index of 3.25 or greater, abnormal respiratory function test, or concerning findings after cardiological evaluation.

Computed tomography (CT) of the chest was used to evaluate the severity of the deformity. The pectus index was calculated as A divided by C, where A is the greatest latero-lateral distance and C is the shortest antero-posterior distance.^3^

**Fig. 3.**
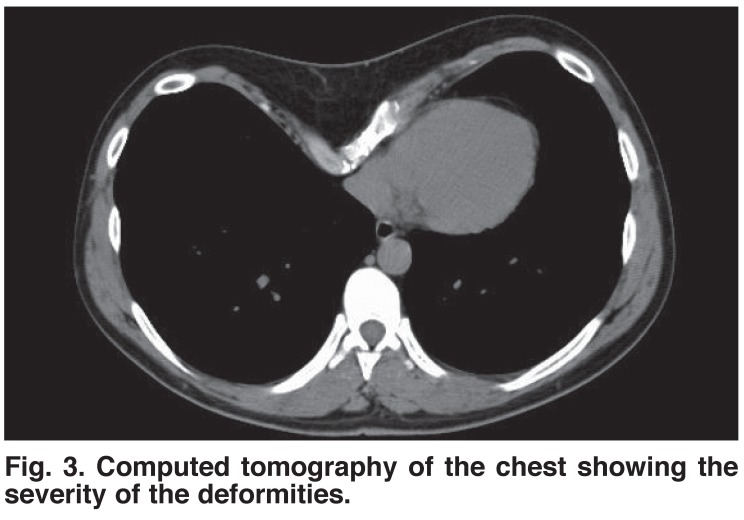
Computed tomography of the chest showing the severity of the deformities.

Chest X-ray, ECG, respiratory function tests and CT were routinely performed on all patients before operating (Fig. 3). All patients were examined by cardiological consultation, and ECG evidence of right ventricular strain was noted in 12% of deformities. Echocardiography showed mitral valve prolapse in 7.3% of the patients.

The Rawitch repair operative technique was used for all 77 patients. General anaesthesia was given and a Foley catheter was placed in all patients. Intravenous cefazolin (1 g) was administered pre-operatively.

A mid-sternal incision was made, and short skin flaps were elevated superiorly and inferiorly using electrocautery. The pectoralis major muscle on each side was then reflected laterally to expose all costal cartilages. The lower costal cartilages are often covered by the rectus muscles.

The deformed costal cartilages were bilaterally resected sub-perichondrially for the full length of the deformed segments. After removal of the costal cartilages, the xiphosternal joint was transacted to enable a finger to pass below the sternum through the mediastinum.

When the pleural space was opened, a small chest tube was inserted for drainage. A transverse wedge osteotomy was made across the anterior sternum where the sternum angled down to the depressed posterior region. The sternum of the posterior region was fractured at the wedge osteotomy without detachment.

Prepared bone or costal cartilage was used to fix the deformity. It was placed in the osteotomy and secured by two transferral monofilament, non-absorbable sutures, and in some cases, a 5-mm stainless steel wire. The perichondrial sheaths were sutured together over the sternal and costal cartilage repair; this is termed plication.

**Fig. 4.**
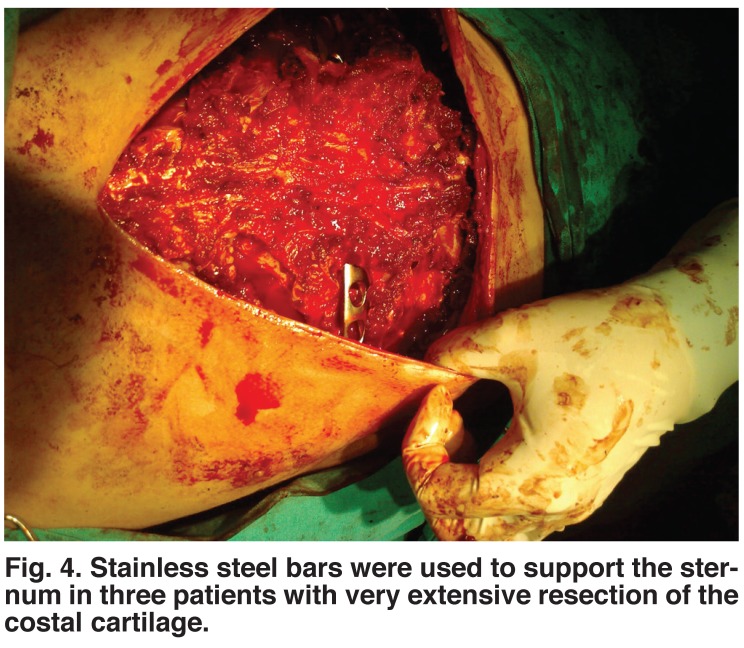
Stainless steel bars were used to support the sternum in three patients with very extensive resection of the costal cartilage.

Haemostasis was achieved with cautery after a haemovac drain had been placed between the muscle layer and the cartilaginous repair. The skin was closed with absorbable subcuticular sutures. For three patients with very extensive resection of the costal cartilage, we used stainless steel bars to support the sternum and prevent flail chest, instead of the standard Kirschner wires (Fig. 4). This method provided excellent mechanical stabilisation of the chest.

In all 77 patients, the endotracheal tube was removed in the operating room. The chest tube and haemovac drain were routinely removed within 48 hours. Intravenous analgesics were giving for the first three postoperative days and then no narcotic medications thereafter. Intravenous cefazolin was given for five additional days. Three patients’ sternal support bar was removed six months later under general anaesthesia.

## Results

A total of 77 patients underwent repair of PE and PC deformities. Their ages ranged from 10 to 22 years (mean 17 years) (Table 1). The mean duration of the operation was 2.5 hours. Mean hospitalisation time was seven days and all patients returned to work or school about 15 days after the operation.

**Table 1:** Age Of Pectus Patients

*Age (years)*	*Number of patients*
10–14	12
14–17	28
17–22	37
Total	77

Before the operation, the most frequent symptoms were aesthetic and cosmetic. Physiological disorders were present in 52%, and other symptoms, which were present to varying degrees, were common: dyspnoea occurred in 20%, and a compression type of discomfort in the lower anterior chest or chest pain occurred in 15%. Palpitations and/or tachycardia were experienced by 8%. Exercise-induced wheezing was reported in 3%, and 2% of patients had an increased frequency of respiratory infections.

Three patients had a sternal bar supporting the sternum using a modified Ravitch technique. One patient had Ehler-Danlos syndrome, and in another we removed a large amount of costal cartilage and the patient needed support to stabilise a flail chest. A third needed extra support because he underwent repair of a second recurrence of PE deformities.

Each of the 77 patients was contacted by telephone, from three months to three years after the operation. Fifteen patients (19%) could not be located and were lost to follow up. In the remaining 62 patients, repair was judged by the patient to be very good or excellent. Each stated he/she would recommend repair of pectus deformities to other patients.

All patients reported marked improvement in exercise tolerance with much less dyspnoea. Most patients were able to participate in exercise such as running, swimming or cycling. All but three patients who had only limited exercise ability before the repair, showed good physical activity six months after the operation. Three patients (3.8%) experienced recurrence, one after PC, and two after PE repair.

## Discussion

The pathophysiology of congenital pectus deformities is poorly understood. It is generally thought that abnormal cartilage growth results in displacement of the sternum either anteriorly in PC or posteriorly in PE.^3^

The majority of children and their parents related to cosmesis and public exposure of the body. A significant number of patients, however, presented with easy fatigue, decreased stamina and wheezing during physical activity, pain and palpitations. Physiological testing is often normal, but both the cardiac index and pulmonary function may be affected, particularly with exercise conditions.^4^

Symptoms of PE and PC are recognised infrequently during early childhood, apart from a child not wanting to go without a shirt while swimming or participating in other athletic or social activities.^5^ Pectus patients commonly try harder to keep up with their peers, using wider diaphragmatic excursions to compensate for diminished chest wall excursions caused by the PE or PC deformity; however, almost all patients experience a worsening of their symptoms with time.

A careful history is important in identifying appropriate operative candidates. Symptoms such as chest pain, palpitations and dyspnoea may indicate underlying cardiac or pulmonary pathology. Inquiring about participation in athletic, physical and social activities provides descriptive information regarding limitations caused by the deformity on cardiopulmonary performance as well as lifestyle.^5^ The deformity gradually worsens, until skeletal growth is complete in late adolescence, and then changes little throughout adult life.

The results of PE repair should be excellent. Peri-operative risks must be limited. The most significant complication is a recurrence, which has been described in a large series as occurring in 5–10% of patients.^6^ A minimal pneumothorax requiring aspiration is infrequent and sometimes needs a thoracostomy tube. Wound infection should be rare with the use of peri-operative antibiotics, especially with protective coverage of the skin during the operative procedure to minimise any contamination by skin flora.^4^

Many improvements in the technique for surgical correction of PE and PC have evolved during the eight decades since the first repairs were performed. Adults with severe pectus deformities and asymmetric defects are at a greater risk of recurrence after a Nuss procedure. These patients may be better repaired initially with a modified Ravitch repair.^7^

A modification of the Ravitch technique for pectus repair was originally described in 1949.2 For example, for PE, the surgical technique includes conservative sub-perichondrial resection of the deformed costal cartilages and detachment of the xiphoid process. The initial steps of the PC correction procedure are similar to that for PE. The sternum, however, is not freed of its environment. A length of 3–4 cm is resected from the distal sternum and the xiphoid process is restored in the proper anatomical direction.^8^

Although the technical aspect of pectus repair is more tedious in adults than in children, postoperative recovery and long-term results have been similar.^9^ The costal cartilages are usually thicker in adults and occasionally they must be scooped out from the perichondrial sheaths with a rongeur rather than being removed with a small elevator. Minimising injury to the perichondrial sheaths during removal of cartilage segments is considered essential in order to permit maximum cartilage regeneration.^10^ Placing minor fragments of fresh autologous cartilage into the empty perichondrial sheaths appears to support costal cartilage regeneration and does not increase the risk of infection.^11^

The Nuss procedure is a minimally invasive technique with a small wound size. The Ravitch procedure provides good correction of pectus deformities. We described here a modification of the Ravitch procedure for PE correction, adding a steel bar under the sternum for support. This procedure has the advantage of preventing postoperative flail chest and mediastinal disorders with severe PE.

Placement of the sternal support across the lower anterior chest appears to provide optimal support for the sternum and may reduce late depression of the upper chest.^12^ In three patients with PE, we noted that placing the bar posterior to the costal cartilages or perichondrial sheaths before attaching it to the appropriate rib on each side elevated the anterolateral chest as well as the sternum to provide optimal cosmetic as well as physiological reconstruction. This technique is used for patients with a higher level of pectus index, who would have developed flail chest with respiratory stress after the postoperative period. It prevents paradoxical respiration, reduces pain, permits early movement, permits deeper inhalation, reduces hospitalisation time and cost, and provides very good long-term results.^13^

Minimal costal cartilage resection provides more stability to the chest during the early postoperative period and more consistent elevation of the anterior chest lateral to the sternum than when more extensive cartilage excision is performed.^3^ Furthermore, there is only moderate postoperative pain, few complications, rapid recovery with early discharge, easy return to physical activities, and consistently good to excellent results compared to the techniques used previously.^14^

Repair of pectus deformities is technically easier and is therefore encouraged during childhood. However, for those patients who have not undergone operations as children, repair during the adult years should be considered by the recommended treatment option of MIRPE (minimal invasive repair of pectus excavatum), as described by Dr Nuss.^15^

MIRPE does not require resection of the cartilage. Firstly the heart and mediastinum are slowly and carefully pushed down by the specific introducer, and the prepared convex steel bar is inserted under the sternum and across the bilateral thoracic incisions. The bar is turned, and the deformed sternum and costal cartilages are then brought into the correct position. The bar is left in place for at least two to three years, depending on the patient and the severity of the deformity.

MIRPE is a minimally invasive procedure with shorter operating time, minimal complication rate, short length of hospitalisation and minimal postoperative pain.^15^ This technique requires neither cartilage incision nor resection.^16^ However, in Turkey, the bar has not been available in state hospitals, which has limited its adoption.

Nowadays, thoracoscopic costal cartilage resection is described for unilateral pectus deformities. This procedure is performed by cutting the anomalous costal cartilages under thoracoscopic imaging, with excellent visibility. The anomalous cartilages are removed with the thoracoscopic view. The anomalous cartilages are identified, and the posterior perichondrium is incised using a hook. Both superior and inferior margins of the cartilage are prepared using a thoracoscopic cautery hook, taking care not to damage the intercostal vessels and nerves.^8^

During the adolescent years of rapid skeletal growth, both PE and PC deformities almost always become much more severe at a time that coincides with increasing athletic activity. The recognition of symptoms and the recommendation for surgical correction remain controversial, with strongly divergent opinions being expressed regarding whether PE and PC are primarily cosmetic disorders, or if they cause physiological impairment and limitations to the patient.

## Conclusion

This retrospective clinical study confirms that pectus deformities can be repaired with a low rate of complications and short hospital stay. The improvement in respiratory symptoms, exercise tolerance and endurance, as well as the cosmetic appearance of more than 97% of the patients in this study supports the view that symptomatic patients of all ages should undergo repair, preferably during the pre-adolescent years.

Routine use of substernal support with minimal pre-operative testing has provided excellent long-term clinical results at a low cost. The most important aspect of pectus correction is to achieve long-term stability of the sternum and thorax after the removal of large amounts of cartilage and sternal osteotomy.16 In three cases, use of a sub-sternal steel bar to stabilise the chest proved advantageous compared to the standard Kirschner wire technique. It is secure, reduces the risk of postoperative complications and provides good cosmetic results.

Kim *et al.* reported successful sub-perichondrial resection, sternal osteotomy and pectus bar insertion placed under the depressed sternum after trauma, followed by internal bar rotation for elevation of the chest wall. This case illustrates that a modified Ravitch procedure using a pectus bar may be an alternative for post-traumatic pectus excavatum.^17^
